# The chromosomal passenger complex controls the function of endosomal sorting complex required for transport-III Snf7 proteins during cytokinesis

**DOI:** 10.1098/rsob.120070

**Published:** 2012-05

**Authors:** Luisa Capalbo, Emilie Montembault, Tetsuya Takeda, Zuni I. Bassi, David M. Glover, Pier Paolo D'Avino

**Affiliations:** 1 Department of Pathology, University of Cambridge, Tennis Court Road, Cambridge CB2 1QP, UK; 2 Cancer Research UK Cell Cycle Genetics Research Group, Department of Genetics, University of Cambridge, Downing Street, Cambridge CB2 3EH, UK

**Keywords:** abscission, Aurora B kinase, Borealin, CHMP4, Shrb

## Abstract

Cytokinesis controls the proper segregation of nuclear and cytoplasmic materials at the end of cell division. The chromosomal passenger complex (CPC) has been proposed to monitor the final separation of the two daughter cells at the end of cytokinesis in order to prevent cell abscission in the presence of DNA at the cleavage site, but the precise molecular basis for this is unclear. Recent studies indicate that abscission could be mediated by the assembly of filaments comprising components of the endosomal sorting complex required for transport-III (ESCRT-III). Here, we show that the CPC subunit Borealin interacts directly with the Snf7 components of ESCRT-III in both *Drosophila* and human cells. Moreover, we find that the CPC's catalytic subunit, Aurora B kinase, phosphorylates one of the three human Snf7 paralogues—CHMP4C—in its C-terminal tail, a region known to regulate its ability to form polymers and associate with membranes. Phosphorylation at these sites appears essential for CHMP4C function because their mutation leads to cytokinesis defects. We propose that CPC controls abscission timing through inhibition of ESCRT-III Snf7 polymerization and membrane association using two concurrent mechanisms: interaction of its Borealin component with Snf7 proteins and phosphorylation of CHMP4C by Aurora B.

## 2. Introduction

Cytokinesis, the final separation of two daughter cells at the end of cell division, requires the coordinated action of several proteins that promote the formation and ingression of a cleavage furrow that bisects the dividing cell. Many key cytokinesis proteins localize to an array of anti-parallel and interdigitating microtubules known as the central spindle, which forms between the segregating anaphase chromosomes during furrow ingression [1]. The central spindle and its associated proteins control furrow formation and ingression, the transport of membrane vesicles to the cleavage site and the final abscission of the two daughter cells [2–4]. In the final stages of cytokinesis, the central spindle forms a compacted structure known as the midbody, which contains at its centre a phase-dense structure—the midbody ring or Flemming body—important for cell abscission. Our knowledge of the mechanisms that control furrow formation and ingression has advanced considerably in the last few years [5]. However, the mechanics and regulation of abscission are still unclear. Recent studies indicate that the endosomal sorting complexes required for transport (ESCRTs) play a key role during cell abscission [6–11]. These evolutionarily conserved complexes are best known for catalysing the sorting of receptors at endosomal membranes into vesicles that bud off into the lumen of the endosome, creating the multivesicular bodies (MVBs) [12]. The cargo of MVBs is then routed into lysosomes for degradation. Four distinct ESCRTs—known as ESCRT-0, -I, -II and -III—are sequentially recruited to endosomes through both protein and lipid interactions [12]. ESCRT-III, the final complex in the pathway, provides the core machinery that mediates membrane deformation and fission events during MVB biogenesis [13]. This complex comprises 11 subunits in humans (also known as charged multivesicular body proteins, CHMPs), which correspond to six counterpart proteins in yeast and seven in *Drosophila* (table 1) [14]. ESCRT proteins have also been implicated in other membrane-budding processes, including retroviral budding and, importantly, cytokinesis that are topologically similar to the budding required for MVB formation [12,14,15]. ESCRT-I and -III components have been found to localize to the midbody, and ESCRT-III components have been proposed to mediate membrane fission at the end of cytokinesis [6–11].

**Table 1.** List of ESCRT-III components in different organisms.

*Saccharomyces cerevisiae*	*Drosophila*	*Homo sapiens*
Vps2/Did4	CG4618	CHMP2A, B
Vps20	Vps20	CHMP6
Vps24	CG9779	CHMP3
Vps32/Snf7	Shrb	CHMP4A, B, C
Vps46	Chmp1	CHMP1A, B
Vps60	CG6259	CHMP5
none	CG5498	CHMP7

The spatial and temporal signals that control abscission in metazoans are poorly understood. One signalling component that has been implicated in these late stages of cytokinesis is the chromosomal passenger complex (CPC). This evolutionarily conserved complex comprises four components: the inner centromeric protein (INCENP), the Aurora B serine/threonine kinase, Survivin and Borealin [16,17]. CPC is essential for multiple processes during cell division, including condensation of chromosomes, their alignment and proper attachment to spindle microtubules, and cytokinesis [17]. Consistent with these functions, the CPC follows a dynamic distribution during mitosis: it localizes to centromeres at mitotic entry, translocates to the central spindle and cleavage furrow after anaphase onset and concentrates at the midbody in late telophase [16]. Studies in many organisms have revealed that CPC components are crucial for completion of cytokinesis [17] and evidence in both yeast and mammals have indicated that Aurora B activity is necessary to prevent cell abscission in the presence of chromosome bridges at the cleavage site, functioning as a ‘NoCut’ checkpoint [18–20].

Here, we show that Borealin interacts directly with Snf7/Shrb/CHMP4 components in both *Drosophila* and human cells and that the two proteins colocalize at the midbody in late cytokinesis. Moreover, we found that Aurora B phosphorylates CHMP4C at three serine residues located in its C-terminal linker region, a part of the protein known to regulate its ability to form polymers and interact with the membrane. Finally, over-expression of CHMP4C variants mutated in these three residues caused cytokinesis failure, suggesting that Aurora B inhibits CHMP4C activity during cytokinesis. We propose that CPC controls abscission timing in both flies and human cells by regulating the function of ESCRT-III Snf7 proteins during cytokinesis through the interaction of its Borealin component with the N-terminus of Shrb/CHMP4 proteins and Aurora B-mediated phosphorylation of the CHMP4C regulatory linker tail.

## 3. Results

### 3.1. Borealin-related protein interacts with the ESCRT-III Shrb component in *Drosophila* cells

**Figure 1.**
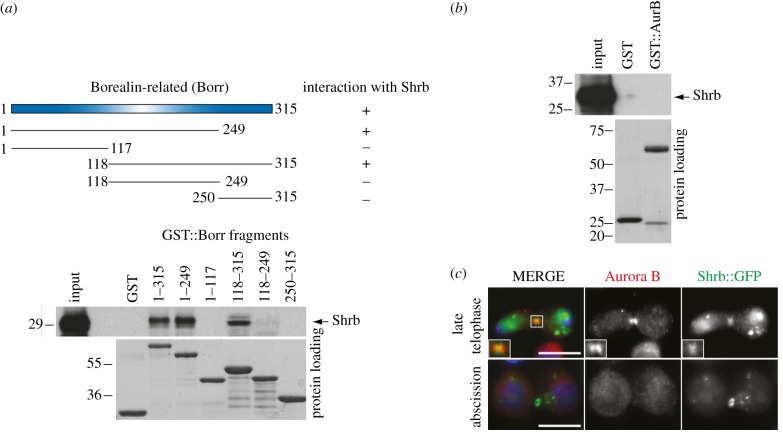
Borr interacts and colocalizes with the ESCRT-III component Shrb in *Drosophila* cells. (*a*) The GST::Borr fragments indicated on the schematic in the upper part were purified from bacteria and incubated with Shrb translated and radio-labelled *in vitro*, and then pulled down using glutathione beads. The Ponceau S staining of the protein loading is shown in the lower part. The numbers on the left indicate the sizes (kDa) of the molecular mass marker. On the right is shown a schematic of the positive (+) and negative (−) results of the GST pull down. (*b*) GST::Aurora B (AurB) was purified from bacteria and incubated with Shrb translated and radio-labelled *in vitro*, and then pulled down using glutathione beads. The Ponceau S staining of the protein loading is shown at the bottom. The numbers on the left indicate the sizes (kDa) of the molecular mass marker. (*c*) *Drosophila* S2 cells stably expressing Shrb::GFP were fixed and stained to detect Aurora B (red in the merged panel), GFP (green in the merged panel) and DNA (blue in the merged panel). Midbody presence and DNA condensation were used as criteria to stage cells during cytokinesis. The inset shows a 2× magnification of the midbody. Scale bar, 10 µm.

In a proteomic survey of complexes involved in cell division in *Drosophila*, we tagged the CPC Borealin-related (Borr) component with two IgG-binding domains of Protein A (PtA) at either its N- or C-terminus. The presence of a tag at either end of Borr did not affect the localization of the protein (see electronic supplementary material, figure S1, and data not shown). Independent *Drosophila* cell lines stably expressing PtA-tagged Borr proteins were generated, and interacting partners isolated by affinity purification and identified by mass spectrometry (MS). In both purifications, we identified the ESCRT-III component Shrb with a score comparable to or even higher than INCENP (table 2). A reciprocal affinity purification using cells expressing PtA::Shrb also identified all the CPC components (table 2), confirming the association *in vivo*. Because Borr and INCENP interact directly, these MS data suggested that Borr could also bind to Shrb directly. To confirm this, we used an *in vitro* glutathione S-transferase (GST) pull-down assay (figure 1*a*). We generated several GST-tagged Borr fragments in bacteria and tested their ability to pull down a radio-labelled Shrb polypeptide synthesized by *in vitro* translation (figure 1*a*). Full-length Borr_1–315_ and the truncated Borr_1–249_ fragment pulled down Shrb very efficiently, whereas Borr_1–117_ did not (figure 1*a*). Borr_118–315_ could also efficiently pull down Shrb, whereas both Borr_118–249_ and the most C-terminal Borr_250–315_ fragment did not bind to Shrb (figure 1*a*). We conclude that the Borr_118–249_ central region is necessary, but not sufficient, for the interaction with Shrb. No direct interaction was observed with two other components of the CPC, INCENP (data not shown) and Aurora B (figure 1*b*), using a similar assay. To assess if and when CPC colocalized with Shrb during mitosis, we generated a stable cell line stably expressing Shrb tagged with green fluorescent protein (GFP). In late telophase, both Shrb::GFP and Aurora B accumulated at the midbody where the two proteins partially colocalized (figure 1*c*). However, Shrb::GFP appeared to localize to the midbody ring, whereas Aurora B was more broadly distributed. Finally, during abscission, only Shrb was visible at the midbody (figure 1*c*). Altogether, these results indicate that Shrb and Borealin interact in late cytokinesis just before abscission.

**Table 2.** Summary of mass spectrometry results from the affinity purifications of PtA-tagged Borr and Shrb. Some of the proteins identified from the affinity purifications are listed along with their relative Mascot score and number of peptides. Deterin is the *Drosophila* orthologue of Survivin.

Bait	interactors	score	peptides
Borr::PtA	Shrb (Snf7)	1099	32
	INCENP	1085	43
	Aurora B	588	26
	Deterin (Survivin)	406	18
PtA::Borr	Shrb (Snf7)	1125	31
	INCENP	714	34
	Aurora B	200	10
	Deterin (Survivin)	333	10
PtA::Shrb	Aurora B	264	10
	INCENP	236	7
	Deterin (Survivin)	204	3
	Borr	48	2

### 3.2. Borealin interacts with ESCRT-III CHMP4 proteins in human cells

**Figure 2.**
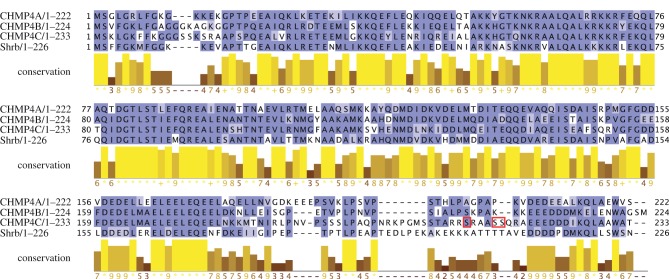
Human and *Drosophila* ESCRT-III Snf7 proteins are very well conserved except in their C-terminal tails. CHMP4A, CHMP4B, CHMP4C and Shrb sequences were aligned using the blast program (http://www.ncbi.nlm.nih.gov/) and the Blosum62 colouring scheme (matches are highlighted in dark blue, and positive alignment scores in light blue). The conservation histogram (yellow and brown bars) is shown below. Conservation is measured as a numerical index reflecting the conservation of physico-chemical properties in the alignment: identities (indicated by asterisks) score highest and the next most-conserved group contains substitutions to amino acids lying in the same physico-chemical class. The serine residues phosphorylated by Aurora B are boxed in red.

**Figure 3.**
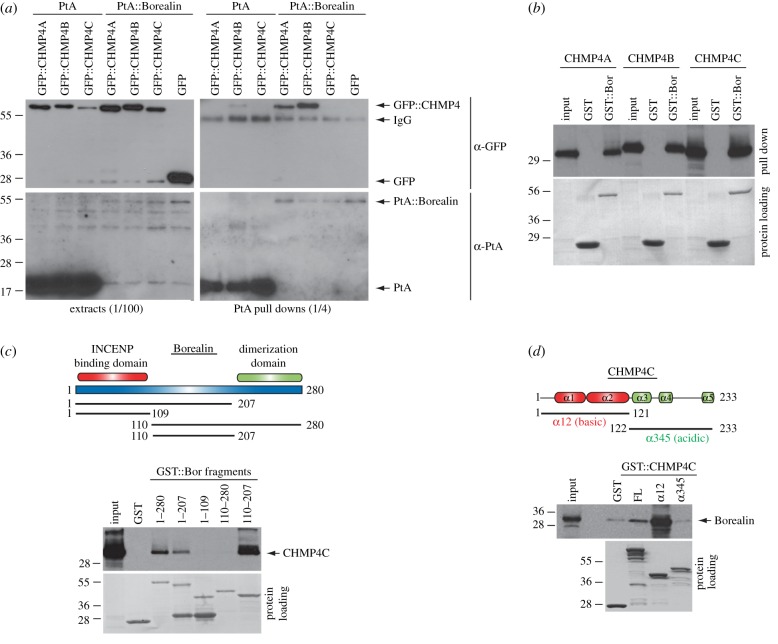
Borealin interacts with ESCRT-III CHMP4 proteins *in vivo* and *in vitro*. (*a*) HeLa cells were transfected with constructs expressing PtA::Borealin or PtA alone and one of the plasmids expressing GFP-tagged CHMP4 proteins or GFP alone. After 48 h, cells were harvested, and protein extracts used in a PtA pull-down assay. The extracts and pull downs were then analysed by Western blot to detect GFP (α-GFP) and PtA (α-PtA). The numbers on the left indicate the sizes (kDa) of the molecular mass marker. (*b*) GST::Borealin (GST::Bor) was purified from bacteria and incubated with CHMP4A, CHMP4B or CHMP4C translated and radio-labelled *in vitro* and then pulled down using glutathione beads. The Ponceau S staining of the protein loading is shown at the bottom. The numbers on the left indicate the sizes (kDa) of the molecular mass marker. (*c*) The schematic in the upper part illustrates Borealin protein domains and the position of different fragments used in the GST pull-down assay. GST::Borealin (GST::Bor) fragments were purified from bacteria and incubated with CHMP4C translated and radio-labelled *in vitro*, and then pulled down using glutathione beads. The Ponceau S staining of the protein loading is shown at the bottom. The numbers on the left indicate the sizes (kDa) of the molecular mass marker. (*d*) The schematic in the upper part illustrates CHMP4C protein domains and the two fragments used in the GST pull-down assay. GST::CHMP4C fragments were purified from bacteria and incubated with Borealin translated and radio-labelled *in vitro* and then pulled down using glutathione beads. The Ponceau S staining of the protein loading is shown at the bottom. The numbers on the left indicate the sizes (kDa) of the molecular mass marker.

We then investigated whether the interaction between Borealin and ESCRT-III Snf7 components had been conserved in human cells. There are three Snf7 paralogues in humans: CHMP4A, CHMP4B and CHMP4C (table 1). The primary sequence of the three CHMP4 proteins and their *Drosophila* homologue Shrb is very well conserved, although all four proteins diverge considerably in their C-terminal regions (figure 2). To assess whether Borealin could interact with CHMP4 proteins *in vivo*, we transfected HeLa cells with either PtA-tagged Borealin or PtA alone as control, and each of the three CHMP4 proteins tagged with GFP. PtA::Borealin pulled down GFP::CHMP4A and GFP::CHMP4B very efficiently, but not GFP::CHMP4C or GFP alone (figure 3*a*). To investigate whether these interactions were direct, we carried out an *in vitro* GST pull-down assay as described earlier for Borr and Shrb. GST-tagged Borealin purified from bacteria could efficiently pull down all three CHMP4 proteins synthesized by *in vitro* translation (figure 3*b*). Our inability to detect an interaction between Borealin and CHMP4C *in vivo*, in contrast with the positive outcome of the *in vitro* assay, suggests that the interaction might be regulated by post-translation modifications and/or may occur only in a specific phase of the cell cycle and therefore, not be easily detectable in our unsynchronized cell populations.

To identify the Borealin domain responsible for the interaction with CHMP4 proteins, we tested the ability of different GST–Borealin fragments to pull down one of the three CHMP4 paralogues. Although all three CHMP4 proteins could strongly bind to Borealin *in vitro,* CHMP4C appeared to interact slightly more efficiently (figure 3*b*) and thus we decided to use this paralogue for the next pull-down assays. Borealin contains an N-terminal region (amino acid 1–109) that interacts with INCENP, and a C-terminal region (amino acid 208–280) required for dimerization (figure 3*c*) [21,22]. We found that the Borealin central region encompassing residues 110–207 was both necessary and sufficient to bind to CHMP4C (figure 3*c*). However, the interaction was lost if the fragment also contained the dimerization domain (figure 3*c*). We conclude that Borealin interacts with CHMP4 proteins via its central region, similar to its *Drosophila* counterpart Borr, but for reasons that are not clear the dimerization domain seems to interfere with the binding ability of the isolated central region but not of the full-length protein (figure 3*c*).

To pinpoint the CHMP4C domain able to bind to Borealin, we tested whether the N- and C-terminal halves of the protein were able to pull down radio-labelled Borealin synthesized by *in vitro* translation (figure 3*d*). These two regions are quite different in their structure and charge. The N-terminal half (amino acid 1–121) is basic and contains two alpha-helices (α12), whereas the C-terminal half (amino acid 122–233) is acidic and contains three alpha-helices (α345; figure 3*d*). We found that GST::CHMP4C_1–121_ could bind to Borealin very efficiently, even better than the full-length protein, whereas GST::CHMP4C_122–233_ could only weakly pull down Borealin to a level similar to that of GST alone (figure 3*d*). Together, these results indicate that the central region of Borealin interacts with the N-terminal basic region of CHMP4C.

**Figure 4.**
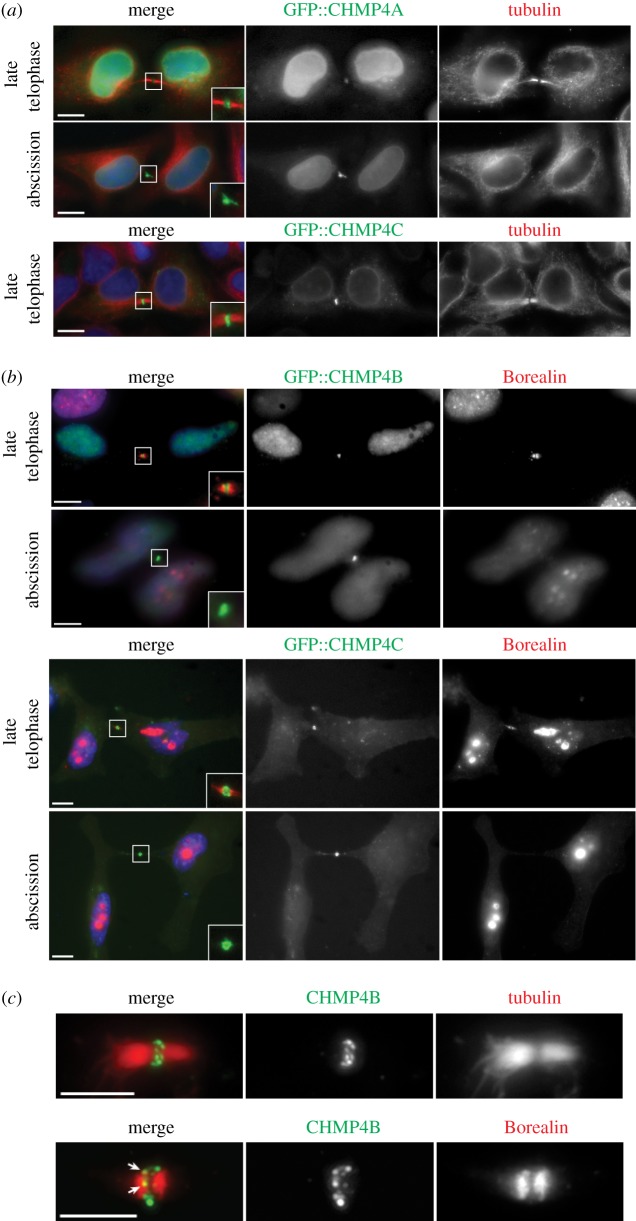
CHMP4 proteins colocalize with Borealin to the midbody in HeLa cells. (*a*) HeLa cells were transfected with constructs expressing GFP::CHMP4A or GFP::CHMP4C for 48 h and then fixed and stained to detect tubulin (red in the merged panels), GFP (green in the merged panels) and DNA (blue in the merged panels). The insets show 2× magnification of the midbody. Scale bars, 10 µm. The presence and thickness of microtubule bundles at the intercellular bridge were used as criteria to stage cells during cytokinesis. (*b*) HeLa cells were transfected with constructs expressing GFP::CHMP4B or GFP::CHMP4C for 48 h and then fixed and stained to detect Borealin (red in the merged panels), GFP (green in the merged panels) and DNA (blue in the merged panels). Midbody presence and DNA condensation were used as criteria to stage cells during cytokinesis. The insets show magnification of the midbody region at 2.5× (GFP::CHMP4B) and 3× (GFP::CHMP4C). Scale bars, 10 µm. (*c*) Midbodies were purified from HeLa cells and then fixed and stained to detect CHMP4B (green in the merged panel) and either tubulin or Borealin (red in the merged panel). The arrows mark the CHMP4B dots that colocalize with Borealin. Scale bars, 5 µm.

We then asked whether Borealin colocalized with CHMP4 proteins during cytokinesis in HeLa cells. We found that all three GFP-tagged CHMP4 proteins accumulated at the midbody in late cytokinesis (figure 4), in agreement with published observations [9,10]. However, both GFP::CHMP4A and GFP::CHMP4B were also found in the nucleus, whereas GFP::CHMP4C was predominantly cytoplasmic (figure 4*a*,*b*). Both GFP::CHMP4B and GFP::CHMP4C partially colocalized with Borealin during late cytokinesis (figure 4*b*) but during abscission only CHMP4 proteins were found at the midbody (figure 4*b*), a localization pattern similar to that observed in *Drosophila* cells (figure 1*c*). To analyse the localization of CHMP4 proteins in more detail, we stained midbodies purified from HeLa cells with antibodies directed against CHMP4B and either tubulin or Borealin (figure 4*c*). These experiments confirmed that CHMP4B accumulated at the midbody and also showed that the ESCRT-III component localized as discrete dots around the Flemming body to form a structure resembling a pearl necklace (figure 4*c*). Some of the CHMP4B dots appeared to colocalize with Borealin (figure 4*c*, arrows). In conclusion, CPC and ESCRT-III Snf7 components associate at the midbody before abscission in both *Drosophila* and human cells.

### 3.3. Aurora B phosphorylates three serine residues in the C-terminal linker region of CHMP4C

**Figure 5.**
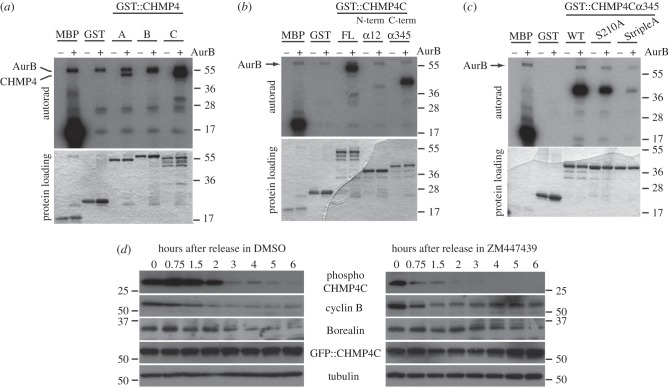
Aurora B phosphorylates CHMP4C *in vitro* and *in vivo*. (*a*) GST-tagged CHMP4 proteins, GST alone or the positive control MBP (myelin basic protein) were incubated with (+) or without (−) recombinant Aurora B in the presence of [γ-^32^P] ATP. The reactions were then separated by SDS-PAGE, and gels stained with Coomassie Blue, dried and exposed at −80°C. The Coomassie Blue staining of the protein loading is shown at the bottom. Note that Aurora B is auto-phosphorylated and co-migrated with GST::CHMP4B. The numbers on the right indicate the sizes (kDa) of the molecular mass marker. (*b*) GST-tagged full-length CHMP4C (FL), GST-tagged N-terminal CHMP4Cα12, GST-tagged C-terminal CHMP4Cα345, GST alone and the positive control MBP were incubated with (+) or without (−) recombinant Aurora B in the presence of [γ-^32^P] ATP. Products of the reactions were then separated by SDS-PAGE and the gels stained with Coomassie Blue, dried and exposed at −80°C. The Coomassie Blue staining of the protein loading is shown at the bottom. The numbers on the right indicate the sizes (kDa) of the molecular mass marker. (*c*) GST-tagged wild-type CHMP4Cα345 (WT), the two GST:: CHMP4Cα345 variants containing S to A mutations at position 210 (S210A) or at position 210, 214 and 215 (StripleA), GST alone and the positive control MBP were incubated with (+) or without (−) recombinant Aurora B in the presence of [γ-^32^P] ATP. Products of the reactions were then separated by SDS-PAGE and the gels stained with Coomassie Blue, dried and exposed at −80°C. The Coomassie Blue staining of the protein loading is shown at the bottom. The numbers on the right indicate the sizes (kDa) of the molecular mass marker. (*d*) HeLa cells were transfected with GFP::CHMP4C, synchronized in metaphase with thymidine/nocodazole block and then released into medium containing either ZM447439 or its solvent DMSO as control. Proteins were extracted, separated by SDS-PAGE, transferred onto PVDF membranes and analysed by Western blot to detect the variant of CHMP4C phosphorylated at serine 210, 214 and 215 (phospho-CHMP4C), cyclin B, Borealin, GFP::CHMP4C and tubulin as loading control. The numbers indicate the sizes (kDa) of the molecular mass marker.

One obvious implication of the interaction between Borealin and CHMP4 proteins is that CPC could regulate ESCRT-III activity through phosphorylation of one or more of its components. To test this hypothesis, we investigated whether Aurora B could phosphorylate recombinant GST::CHMP4 proteins in an *in vitro* kinase assay. We found that all three GST::CHMP4 proteins were phosphorylated by Aurora B *in vitro*, although GST::CHMP4C seemed the best target (figure 5*a*). Therefore, we next focused on the identification of the CHMP4C residues phosphorylated by Aurora B using the same *in vitro* phosphorylation assay. Only the acidic C-terminal half of CHMP4C was strongly phosphorylated by Aurora B (figure 5*b*), and an *in silico* prediction analysis using the GPS 2.1 software [23] identified a serine located at position 210 as the best candidate for phosphorylation by Aurora B (table 3). The introduction of a serine (S) to alanine (A) mutation at this position (S210A) significantly reduced, but did not completely abolish, the level of Aurora B phosphorylation (figure 5*c*). To identify the other Aurora B phosphorylation sites, we mutagenized the majority of serines and threonines listed in table 3 to alanine and analysed the effect of these mutations, either alone or in combination with S210A, on Aurora B phosphorylation (see electronic supplementary material, figure S2). The introduction of the S214A and S215A mutations together with S210A almost completely abolished Aurora B phosphorylation (figure 5*c* and see electronic supplementary material, figure S2) indicating that these three residues are the major Aurora B phosphorylation sites in the C-terminal region of CHMP4C. Quite interestingly, these residues lie in a ‘linker’ region between the last two alpha-helices that has been shown to be important for the regulation of CHMP4 protein activity [24].

**Table 3.** Summary of the results from *in silico* prediction of potential Aurora B phosphorylation sites in the C-terminal half of CHMP4C and their validation by Aurora B *in vitro* phosphorylation assay.

position	residue	peptide	GPS 2.1 score	*in vitro* phosphorylation
136	T	DDLMQEITEQQDIAQ	0.931	nd
146	S	QDIAQEISEAFSQRV	1.621	no
150	S	QEISEAFSQRVGFGD	0.414	no
181	T	EELNKKMTNIRLPNV	2.793	no
190	S	IRLPNVPSSSLPAQP	0.207	no
191	S	RLPNVPSSSLPAQPN	0.483	no
192	S	LPNVPSSSLPAQPNR	2.069	no
204	S	PNRKPGMSSTARRSR	1.724	no
205	S	NRKPGMSSTARRSRA	2.276	no
206	T	RKPGMSSTARRSRAA	0.655	nd
210	S	MSSTARRSRAASSQR	10.379	yes
214	S	ARRSRAASSQRAEEE	2.448	yes
215	S	RRSRAASSQRAEEED	2.655	yes
233	T	KQLAAWAT	0.655	nd

To analyse CHMP4C phosphorylation *in vivo*, we generated an antibody against a CHMP4C peptide encompassing residues 206–220 and containing phosphorylated serines at positions 210, 214 and 215 (TARR*S*RAA*SS*QRAEEC). This antibody detected a band of approximately 30 kD and a band corresponding to GFP-tagged CHMP4C in Western blots of synchronized HeLa cell extracts. Both signals were absent in a twin blot that had been pre-incubated with λ-phosphatase (see electronic supplementary material, figure S3), indicating that our antibody specifically recognizes a phosphorylated form of CHMP4C. We next used this antibody to analyse the profile of phosphorylated CHMP4C during mitotic exit and after treatment with the Aurora B inhibitor ZM447439 [25]. HeLa cells were transfected with GFP::CHMP4C, synchronized in metaphase by thymidine/nocodazole block and then released into medium containing either ZM447439 or its solvent DMSO as control. In control cells, phosphorylated CHMP4C was strongly reduced 3 h after nocodazole release, immediately after cyclin B degradation and concomitantly with a reduction in the level of Borealin (figure 5*d*). These results indicate that CHMP4C is dephosphorylated during mitotic exit at a time when cells have already completed furrowing and CPC is being degraded. We rule out the possibility that this profile might reflect overall degradation of CHMP4C because the signal relative to GFP::CHMP4C (detected with an anti-GFP antibody) did not follow the same profile as the signal detected by our phospho-specific antibody (figure 5*d*). Unfortunately, we could not analyse the expression of endogenous CHMP4C because an antibody we generated against the non-phosphorylated CHMP4C peptide and other two commercially available antibodies could detect only the over-expressed protein and not the endogenous pool of CHMP4C. Strikingly, the phospho-specific CHMP4C signal disappeared much more rapidly after the addition of the Aurora B inhibitor, even though both cyclin B and Borealin persisted (figure 5*d*). We therefore conclude that Aurora B phosphorylates CHMP4C *in vivo* and that the Aurora B target sites are dephosphorylated in cells that have completed furrow ingression.

### 3.4. CHMP4C phospho-mimic and non-phosphorylatable mutants accumulate normally at the midbody but their over-expression cause cytokinesis failure

**Figure 6.**
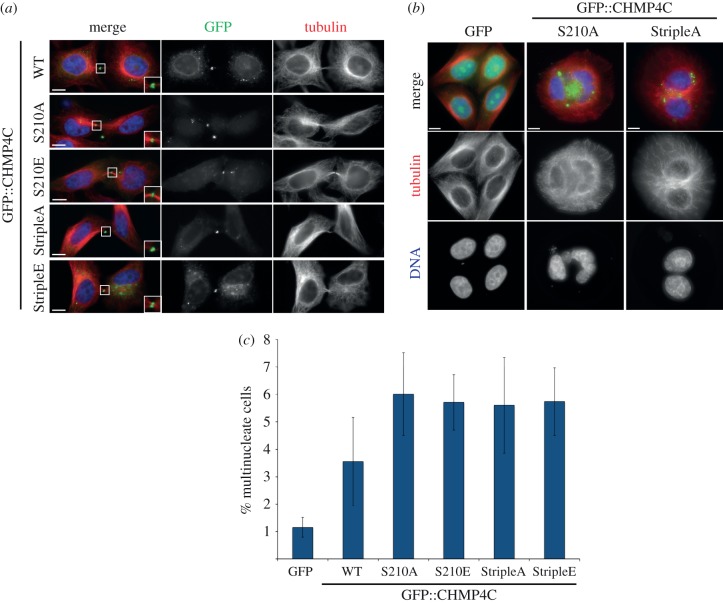
Over-expression of phospho-mimic and non-phosphorylatable CHMP4C mutants leads to cytokinesis failure. (*a*) HeLa cells were transfected with constructs expressing wild-type GFP::CHMP4C (WT) or one of the GFP::CHMP4C variants containing S to A or S to E substitutions at position 210 (S210A and S210E) or at position 210, 214 and 215 (StripleA and StripleE) for 48 h and then fixed and stained to detect tubulin (red in the merged panel), GFP (green in the merged panel) and DNA (blue in the merged panel). The insets show 2× magnification of the midbody. Scale bars, 10 µm. (*b*) Examples of multinucleate cells obtained after transfection with GFP::CHMP4C mutants. HeLa cells were transfected with constructs expressing one of the GFP::CHMP4C variants containing S to A substitutions at position 210 (S210A) or at position 210, 214 and 215 (StripleA) or GFP alone as a control for 48 h and then fixed and stained to detect tubulin (red in the merged panel), GFP (green in the merged panel) and DNA (blue in the merged panel). Note that the cells expressing the two GFP::CHMP4C mutants are multinucleated. Scale bars, 10 µm. (*c*) Percentage of multinucleate cells after treatment for 48 h with the GFP::CHMP4C constructs shown in panel (*a*). At least 600 cells were counted in each experiment, *n* = 4. Bars indicate standard errors.

To investigate whether Aurora B phosphorylation could affect CHMP4C localization and/or function, we transfected HeLa cells with GFP::CHMP4C mutants containing either phospho-mimic (serine to glutamate; S to E) or non-phosphorylatable (S to A) substitutions at position 210 alone or at positions 210, 214 and 215 (triple mutants). All the mutants accumulated at the midbody with a pattern similar to the wild-type protein (figure 6*a*). This suggests that the phosphorylation status of CHMP4C does not affect its ability to accumulate at the midbody, with the caveat that phospho-mimic mutants might not exactly reflect the localization and behaviour of the endogenous pool of phosphorylated CHMP4C. We next asked if expression of these GFP::CHMP4C mutants could affect cytokinesis. Over-expression of GFP-tagged wild-type CHMP4C has already been reported to cause cytokinesis failure [6] and we found that transfection of all four GFP::CHMP4C phospho-mutants caused an increase in the number of multi-nucleated cells compared with control cells transfected with GFP alone or cells transfected with wild-type GFP::CHMP4C (figure 6*b*,*c*). That the presumptive phospho-mimic mutants behaved like the non-phosphorylatable variant might indicate either that the S to E substitutions cannot successfully mimic Aurora B phosphorylation or that the CHMP4C phosphorylation–dephosphorylation cycle must be tightly regulated for proper CHMP4C function. Taken together, our results indicate that phosphorylation by Aurora B controls the function of CHMP4C during late cytokinesis.

## 4. Discussion

**Figure 7.**
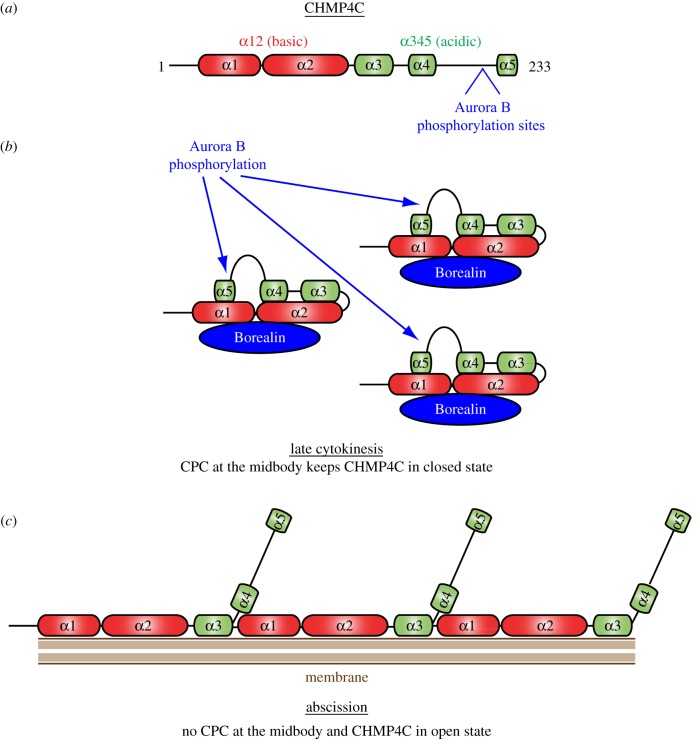
Model for Aurora B-mediated regulation of CHMP4C during cytokinesis. (*a*) Schematic of CHMP4C indicating the position of the Aurora B target sites. (*b*) Interaction with Borealin and phosphorylation by Aurora B keep CHMP4C in its ‘closed’ state at the midbody before abscission. (*c*) When CPC is no longer at the midbody, CHMP4C can convert into its ‘open’ state and form membrane-associated polymers that mediate abscission.

Our findings identify a novel, evolutionarily conserved interaction between CPC and ESCRT-III Snf7 components. In both *Drosophila* and human cells, the CPC Borealin subunit interacts directly with Shrb/CHMP4 proteins, and in human cells Aurora B phosphorylates the C-terminal tail of CHMP4C. The latter mechanism seems to have evolved specifically for the human CHMP4C protein because the three residues phosphorylated by Aurora B—S210, S214 and S215—are not present in the other two human paralogues and Shrb (figure 2). Consistent with this, Aurora B weakly phosphorylated CHMP4A, CHMP4B and Shrb *in vitro* (figure 5, and data not shown). The lack of conservation of phosphorylation sites between orthologues is not uncommon; for example, the Polo-like kinase-1 phosphorylation sites in PRC1, a protein required for bundling the central spindle, are not conserved in its *Drosophila* orthologue Fascetto [26,27]. Our results suggest that the CHMP4C phosphorylation status does not affect its ability to accumulate at the midbody but it does impair its function during cytokinesis (figure 6). Interestingly, the serines phosphorylated by Aurora B are located in a region linking the C-terminal-most two alpha-helices of CHMP4C (figure 7). This region has been found to be essential for regulating the activity of many ESCRT-III proteins in various biological processes, including viral budding and cytokinesis [8,24]. Current models propose that ESCRT-III proteins cycle between a default auto-inhibitory or ‘closed’ status and an ‘open’, active form (figure 7) [24,28]. In the ‘closed’ status, the basic and acidic halves of the ESCRT-III protein are tightly associated, and the protein is soluble and does not interact with other components of the ESCRT machinery and the membrane. In the ‘open’ form, the two halves of the ESCRT-III protein dissociate, and the protein forms polymers that associate with the membrane and other ESCRT-III components (figure 7) [24]. The C-terminal tail of ESCRT-III proteins, including the last α-helix and the linker region, has been proposed to play a key role in the transition from the closed to the open status, possibly through its interaction with other regulatory factors [24,28]. Our results suggest that Aurora B phosphorylation could prevent the conversion of CHMP4C from a closed, auto-inhibitory status into an open, active status (figure 7), possibly by inhibiting the association of CHMP4C with some other regulatory partner(s).

The interaction of Borealin with the N-terminal basic region of CHMP4 components could simply serve to bring Aurora B in close proximity of its CHMP4C target or could have itself a regulatory function. The evidence that this interaction is conserved in *Drosophila* (figure 1), where Shrb does not contain the Aurora B phosphorylation sites identified in CHMP4C (figure 2) and does not seem to be a target of this kinase (data not shown), strongly supports this second hypothesis. Thus, we would like to propose a model where CPC could preclude the activation of CHMP4 components at the cleavage site by two concurrent mechanisms: (i) the binding of Borealin to CHMP4 proteins could directly interfere with their ability to form polymers and associate with the membrane, and (ii) Aurora B phosphorylation could prevent the interaction of CHMP4C with regulatory factors necessary for its transition from an auto-inhibited to an active state (figure 7). These two mechanisms could inhibit the formation of CHMP4 arrays or filaments that have been described at the abscission site in human cells [10] and thus abscission could proceed only when CPC is no longer present at the midbody (figure 7). This model would explain how CPC could control abscission at the end of cytokinesis to prevent cell separation in the presence of DNA at the cleavage site and the consequent formation of aneuploid and/or polyploid cells [18,19].

## 5. Material and methods

### 5.1. Cell culture, DNA transfection and siRNA treatments

The Dmel strain of S2 cells (Invitrogen) was grown in a serum-free medium supplemented with antibiotics. Generation of blasticidin-resistant stable cell lines was performed as described [29] with the only exception that Fugene HD transfection reagent (Roche) was used following the supplier's instructions. Human HeLa (Kyoto and S3) cells were maintained in Dulbecco's modified Eagle medium (Invitrogen) supplemented with 10 per cent foetal calf serum and antibiotics. To synchronize cells in metaphase, HeLa cells were arrested in S phase by the addition of 2 mM thymidine (Sigma) for 18 h, washed twice with phosphate-buffered solution (PBS) and released for 5 h in a fresh complete medium. After release, cells were cultured for a further 16 h in a fresh complete medium in the presence of 50 ng ml^−1^ nocodazole (Sigma) and then harvested by mitotic shake-off. Mitotic cells were washed three times with PBS and resuspended in a fresh complete medium with or without the addition of the Aurora B inhibitor ZM447439 (Tocris Bioscience) at the final concentration of 2 μM.

### 5.2. Midbody purification

HeLa S3 cells were synchronized in metaphase as described earlier, but after nocodazole treatment, they were released in a fresh medium supplemented with 25 μM MG132 (Sigma). After 2 h, cells were washed three times in PBS, released in a normal medium and allowed to progress throughout mitosis. After 1.5–2 h, when the vast majority of cells had completed furrowing, cells were centrifuged at 200*g* for 3 min, washed once with warm H_2_O and gently resuspended in a hypotonic swelling solution containing 1 mM PIPES pH 7, 1 mM MgCl_2_, 5 μg ml^−1^ Taxol (Sigma) and a cocktail of protease inhibitors (Roche). Cells were immediately centrifuged at 200*g* for 3 min, resuspended in a solution containing 1 mM PIPES pH 7, 1 mM EGTA, 1 per cent NP40, 5 μg ml^−1^ Taxol and protease inhibitors (Roche), and vortexed vigorously. After the addition of 0.3 volumes of cold 50 mM 2-(*N*-morpholino) ethane sulphonic acid (MES) pH 6.3, cells were incubated on ice for 20 min and then centrifuged at 250*g* for 10 min. The supernatant was then transferred to a new tube and centrifuged at 740*g* for 20 min to pellet midbodies. The pellet was resuspended in 50 mM MES pH 6.3, layered over a cushion of 40 per cent glycerol (w/v) in 50 mM MES pH 6.3 and centrifuged at 2800*g* for 45 min. After a final wash in 50 mM MES pH 6.3, midbodies were plated on poly-lysine-coated coverslips and processed for immunofluorescence as described in §5.6.

### 5.3. Molecular biology

Gateway technology (Invitrogen) was used in all cloning procedures as described [30]. The destination vectors used for the expression in *Drosophila* cultured cells were described [29–31]. The pDEST15 vector (Invitrogen) was used for bacterial expression of GST-tagged proteins. pDEST53 and pDEST47 (Invitrogen) were used to express GFP-tagged proteins in human cells. pNPE1 (a gift from G. Tzolovsky, University of Cambridge, UK) was used to express PtA-tagged Borealin in HeLa cells.

### 5.4. Affinity purification and *in vivo* pull-down assays

PtA purifications were performed essentially as described previously [29,31]. Approximately, 10^9^ cells were harvested by centrifugation and frozen in liquid nitrogen. The cell pellet was then resuspended in 5 ml of extraction buffer (50 mM HEPES pH 7.5, 100 mM KAc, 50 mM KCl, 2 mM MgCl_2_, 2 mM EGTA, 0.1% NP-40, 5 mM DTT, 5% glycerol and Roche Complete Protease Inhibitors) and homogenized using a high-performance disperser (Fisher). Homogenates were agitated at 4°C for 30 min and clarified by centrifugation at 6000 r.p.m. in a SS34 rotor. Dynabeads (200 µl; Invitrogen) conjugated to rabbit IgG (MP Biochemicals) were added to the supernatants and incubated for 4 h under continuous agitation at 4°C. Beads were then washed five times for 10 min in 10 ml of extraction buffer. Proteins were then eluted from beads with 0.5 M NH_4_OH and 0.5 mM EDTA, concentrated, and analysed by LC–MS/MS. The MS/MS fragmentation data that we achieved were used to search the National Center for Biotechnology Information and Flybase databases using the MASCOT search engine (http://www.matrixscience.com). Probability-based MASCOT scores were used to evaluate identifications. Only matches with *p* < 0.05 for random occurrence were considered significant.

For PtA pull-down assays in HeLa cells, cells were collected and processed as explained earlier. Eluates were then fractioned on an 8–16 per cent SDS–PAGE, transferred onto a polyvinylidene fluoride (PVDF) membrane and probed with the antibodies indicated in figure.

### 5.5. *In vitro* binding assay

DNA fragments coding for Borr, Borealin and CHMP4C were generated by PCR and cloned into pDEST15 (Invitrogen) to express N-terminal GST-tagged polypeptides in *Escherichia coli*. The GST-tagged products were then purified using Glutathione Sepharose 4B according to the manufacturer's instruction (GE Healthcare). [^35^S] Methionine-labelled Borealin, CHMP4A, CHMP4B, CHMP4C and Shrb fragments were prepared from corresponding PCR products amplified using primers harbouring a T7 promoter and then transcribed and translated *in vitro* using the TnT T7 quick-coupled transcription/translation system (Promega) in the presence of [^35^S] methionine (Perkin Elmer). Generally, 25 μl of glutathione sepharose beads containing purified GST-proteins were mixed with 10 μl of [^35^S] methionine-labelled polypeptides and 300 μl of NET-N + buffer (50 mM Tris–HCl, pH 7.4, 150 mM NaCl, 5 mM EDTA, 0.5% NP-40 and a cocktail of Roche complete protease inhibitors) and incubated in ice for 30 min with periodic agitation. The mixture was then washed five times by adding 500 μl of NET-N+ buffer followed by centrifugation at 500*g* for 1 min. Beads were resuspended in 25 μl of Laemmli SDS-PAGE sample buffer and typically one-fifth of the mixture (10 µl) was loaded on 8–16 per cent Tris–Glycine gel. Proteins were then transferred onto a nitrocellulose membrane using the iBlot dry blotting system (Invitrogen) and exposed to X-ray films at −80°C.

### 5.6. Antibodies and microscopy

Antibodies were raised in rabbits against a synthetic CHMP4C peptide encompassing residues 206–220 and containing phosphorylated serines at position S210, S214 and S215 (TARR*S*RAA*SS*QRAEEC). Peptide synthesis, conjugation, rabbit immunizations, serum production and affinity purification were carried out by Abgent (Generon, UK). Other antibodies used in this study are rabbit anti-CHMP4B (a kind gift of A. Sagona and H. Stenmark [32]) mouse monoclonal anti-tubulin (clone DM1A, Sigma), rabbit anti-Aurora B (*Drosophila*; [33]) mouse monoclonal anti-Borealin, mouse monoclonal anti-GFP (Roche), peroxidase-chrompure anti-rabbit IgG (Jackson Laboratories). Peroxidase and Alexa-fluor-conjugated secondary antibodies were purchased from Jackson Laboratories and Invitrogen.

For immunofluorescence, Dmel and HeLa cells were grown on 22 × 22 mm coverslips (Menzel-Gläser) and fixed in PHEM buffer (60 mM Pipes, 25 mM Hepes pH 7, 10 mM EGTA, 4 mM MgCl_2_, 3.7% formaldehyde) for 12 min. They were then washed three times for 10 min with PBS and incubated in blocking buffer (PBS, 0.5% Triton X-100, 3–5% BSA) for 1 h at room temperature. Incubation with primary antibodies was performed over night at 4°C in PBT (PBS, 0.1% Triton X-100, 1% BSA). After two washes in PBT, coverslips were incubated with Alexa-fluor-conjugated secondary antibodies (Invitrogen) diluted in PBT for 2 h at room temperature and then washed twice with PBT and once with PBS. Coverslips were finally mounted using Vectashield mounting medium with DAPI (4′,6-diamidino-2-phenylindole; Vector Laboratories) and visualized on a Zeiss Axiovert 200 fluorescence microscope equipped with a 100× objective (NA 1.4). Images were acquired using a Photometrics CoolSNAP HQ2 camera with MetaMorph software (Molecular Devices) and assembled and adjusted for contrast using Adobe Photoshop CS3.

### 5.7. *In vitro* phosphorylation assay

Purified GST-tagged CHMP4A, CHMP4B and CHMP4C proteins, including CHMP4Cα12, CHMP4Cα345 and various CHMP4Cα345 variants containing S to A mutations, were incubated with or without 100 ng recombinant Aurora B (Cell Signaling) in a kinase buffer (20 mM Hepes pH 7.5, 5 mM MgCl2, 1 mM DTT, 0.1 mM ATP) containing 2 μCi [γ-^32^P]ATP (Perkin Elmer). Reactions were incubated at 30°C for 30 min, quenched by the addition of 2× Laemmli sample buffer, boiled and separated by electrophoresis on a 4–20 per cent SDS-PAGE. Gels were stained with Bio-Safe Coomassie stain (Bio-Rad), and then dried and exposed at −80°C.

### 5.8. Phosphatase treatment

Proteins were separated by SDS-PAGE and transferred to PVDF Immobilon-P membrane (Millipore). The blots were then washed twice with water, blocked in Tris-buffered saline (TBS) containing 0.1 per cent BSA and 0.1 per cent Triton X-100 for 1 h at room temperature and then incubated in phosphatase buffer (TBS 1X, 0.1% BSA, 0.1% Triton X-100, 2 mM MnCl_2_) containing 400 units ml^−1^ of λ-phosphatase (NEB) for 4 h at room temperature. After incubation, blots were washed for 5 min in PBS + 0.1 per cent Tween 20 and processed for antibody detection.

## Supplementary Material

Supplemental Material
