# Supplementary material for: The chromosomal passenger complex controls the function of endosomal sorting complex required for transport-III Snf7 proteins during cytokinesis
Source: Open Biol. 2012 May;2(5):120070. doi: 10.1098/rsob.120070 (PMC3376741; doi:10.1098/rsob.120070)
Supplement: Supplemental Material [file rsob120070-s1.pdf]

Capalbo et al. Supplementary Material

Figure S1. Borr::GFP localization during mitosis in *Drosophila* cells. *Drosophila* S2 cells stably expressing Borr::GFP were fixed and stained to detect GFP (green in the merged panel), tubulin (red in the merged panel) and DNA (blue in the merged panel). Scale bar is 10µm.

Figure S2. Aurora B phosphorylation of various CHMP4C mutants *in vitro*. *(a)* and *(b)*, GST-tagged wild type CHMP4Cα345 (WT), GST::CHMP4Cα345 variants containing S to A mutations at the positions indicated at the top, GST alone and the positive control MBP (myelin basic protein) were incubated with (+) or without (-) recombinant Aurora B in the presence of [γ-^{32}P] ATP. The reactions were then separated by SDS PAGE and the gels stained with Coomassie Blue, dried and exposed at -80ºC. The Coomassie Blue staining of the protein loading is shown at the bottom. The numbers on the right indicate the sizes in kilodaltons of the molecular mass marker.

Figure S3. Validation of the phospho-specific CHMP4C antibody. HeLa cells were transfected with GFP::CHMP4C (+) or a control plasmid (-) and synchronized in metaphase by thymidine/nocodazole block. Proteins were extracted, separated by SDS PAGE and transferred onto PVDF membranes in duplicate. One blot was incubated with λ-phosphatase and then both membranes were processed together to detect the variant of CHMP4C phosphorylated at serine 210, 214 and 215 (phoshpo-CHMP4C) and tubulin as loading control. Note that the phospho-CHMP4C antibody cross-reacted with the molecular ladder, providing an internal control for the experiment.

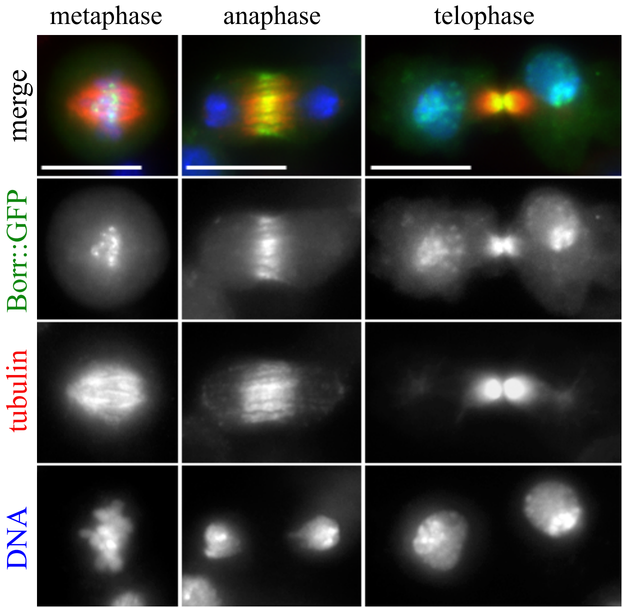

Supplementary figure S1
Capalbo et al.

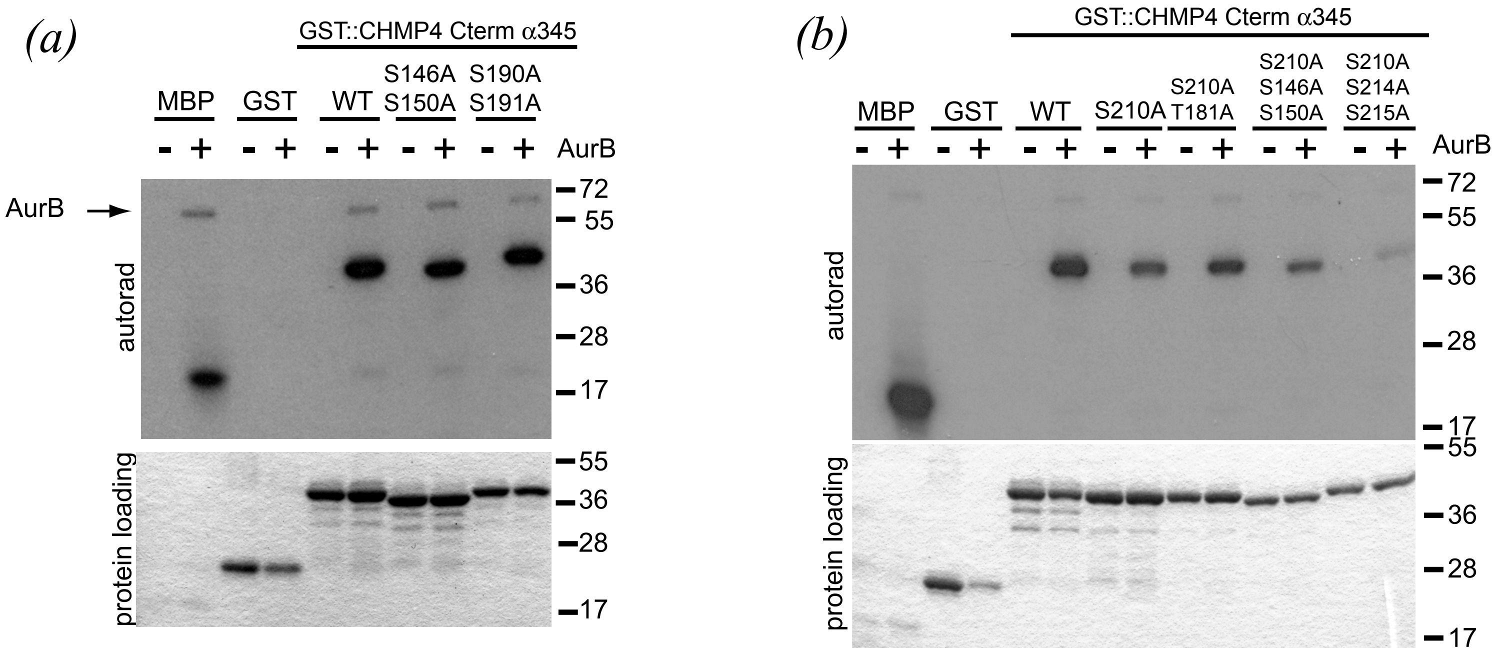

Supplementary figure S2
Capalbo et al.

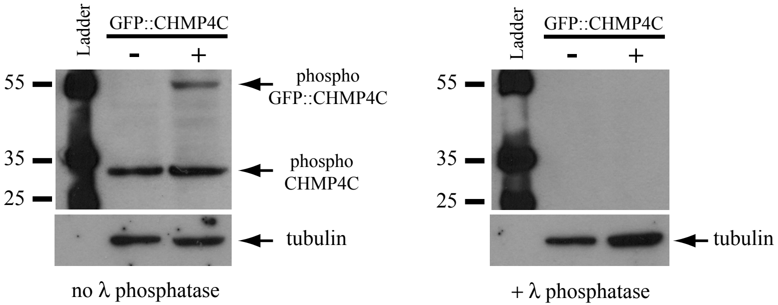

Supplementary figure S3
Capalbo et al.
